# A parasitic plant increases native and exotic plant species richness in vernal pools

**DOI:** 10.1093/aobpla/plv100

**Published:** 2015-08-22

**Authors:** Andrea M. Graffis, Jamie M. Kneitel

**Affiliations:** Department of Biological Sciences, California State University, Sacramento, CA 95819-6077, USA

**Keywords:** *Cuscuta howelliana*, keystone species, parasitic plant, species composition, temporary pond

## Abstract

Species interactions are well known to affect species diversity in communities, but the effects of parasites have been less studied. Previous studies on parasitic plants have found both positive and negative effects on plant community diversity. *Cuscuta howelliana* is an abundant endemic parasitic plant that inhabits California vernal pools. We tested the hypothesis that *C. howelliana* acts as a keystone species to increase plant species richness in vernal pools through a *C. howelliana* removal experiment at Beale Air Force Base in north-central California. Vernal pool endemic plants were parasitized more frequently, and *Eryngium castrense* and *Navarretia leucocephala* were the most frequently parasitized host plant species of *C. howelliana. Cuscuta howelliana* caused higher plant species richness, both natives and exotics, compared with removal plots. However, there was no single plant species that significantly increased with *C. howelliana* removal. Decreases in *Eryngium castrense* percent cover plots with *C. howelliana* is a plausible explanation for differences in species richness. In conclusion, *C. howelliana* led to changes in species composition and increases in plant species richness, consistent with what is expected from the effects of a keystone species. This research provides support for a shift in management strategies that focus on species-specific targets to strategies that target maintenance of complex species interactions and therefore maximize biodiversity and resilience of ecosystems.

## Abstract (precis)

Parasitic plants can have a variety of effects on species diversity in communities. We tested whether Dodder, *Cuscuta howelliana*, acted as a keystone species in California vernal pools. We conducted a host species usage study and a Dodder removal experiment. Vernal pool endemics were more likely to be parasitized and decreased the cover of one species. Dodder presence increased species richness, but this was true for both native and exotic species. Therefore, Dodder acts as a keystone species in California vernal pools

## Introduction

A host of species interactions within and among trophic levels affects ecological community structure. Ecologists are especially interested in the interactions that can result in the promotion of species diversity in communities. For example, keystone predation increases species diversity by the preferential consumption of dominant competitors and thereby prevents the extinction of inferior competitors (Paine 1966; Mills *et al*. 1993). These effects are documented with various predators (Estes and Palmisano 1974; Mills *et al*. 1993), herbivores (Naiman *et al*. 1986; Bowers *et al*. 1987; Sarnelle 2005) and parasites (Pennings and Callaway 1996; Grewell 2008; Watson 2009; Hatcher *et al*. 2012). Parasitic plants have the potential to play the role of a keystone predator in communities since they can reduce the cover of their hosts (e.g. Pennings and Callaway 2002), but they have been less studied in this context.

Thousands of parasitic plant species are found worldwide (Kuijt 1969), which have positive or negative effects on species diversity at multiple scales (Pennings and Callaway 1996; Press and Phoenix 2005). For example, *Rhinanthus minor*, a hemiparasitic plant, decreased diversity of sand dune communities through preferentially parasitizing a competitively inferior species (Gibson and Watkinson 1989; Press and Phoenix 2005). In contrast, the parasitic plant *Cuscuta salina* increased plant diversity in coastal wetlands where their preferred host plant is competitively dominant (Pennings and Callaway 1996; Grewell 2008). This variation in community responses highlights the need to better understand changes in species richness and community composition in response to parasitic plants, especially in highly managed communities.

*Cuscuta howelliana* (Convolvulaceae), or Boggs Lake dodder, is endemic to California vernal pools (Costea and Stefanović 2012) and is an obligate annual vine parasite that has a near full loss of photosynthetic capability (Revill *et al*. 2005). *Cuscuta howelliana* obtains its nutrients through its haustoria that tap into the host plants phloem (Kuijt 1969). It generally parasitizes common vernal pool plants, including *Eryngium castrense* (Apiaceae), *Navarretia leucocephala* (Polemoniaceae), *Polygonum polygloides* subspecies *kelloggi* (Polygonaceae) and *Epibolium campestre* (Onagraceae) (Costea and Stefanović 2012). Little is known, however, about the frequency with which it attacks different hosts and the effects on plant community composition in California vernal pools.

California vernal pools are temporary wetlands that support a diverse community of over 70 endemic plant and invertebrate species (Stone 1990; Keeley and Zedler 1998; Baldwin *et al*. 2012), including many threatened and endangered species (Federal Register 2003). Recent development of 90–97 % of vernal pool habitat has resulted in many endemic vernal pool species becoming endangered (Holland and Jain 1988; Holland 1998; Federal Register 2003). In addition, exotic species invasions have increasingly become problematic for maintaining species diversity in vernal pools (Collinge *et al*. 2011). Vernal pools are also the focus of many management, restoration and mitigation efforts in California (Federal Register 2003). Despite extensive work on plant community structure in this system (Holland and Jain 1981; Bliss and Zedler 1997; Gerhardt and Collinge 2003; Collinge *et al*. 2011; Faist *et al*. 2013), the community effects of the endemic parasitic plant *C. howelliana* has not been investigated.

The goal of this study was to understand the role of *C. howelliana* (hereafter, we will also use Dodder) in California vernal pools. First, the variation in Dodder infection levels within and across vernal pools was determined. Second, a field experiment removed Dodder and measured the response of plant species cover and richness (natives and exotics). If there is a decrease in abundance of a competitive dominant plant due to parasitism by Dodder, this has the potential to develop opportunities for competitively inferior plants. Consequently, Dodder may act as a keystone predator in vernal pools, which would be important for restoration and management of this greatly reduced habitat. Knowledge of the effect of *C. howelliana* on vernal pool plant diversity contributes to a more complete understanding of the role parasitic plants play in shaping community composition.

## Methods

### Study system

This study took place at Beale Air Force Base (Yuba County, CA, USA), located in northeastern Sacramento Valley at the foot of the Sierra Nevada mountain range. There are ∼1000 vernal pools within Beale Air Force Base, and the elevation ranges from 24 to 180 m. Vernal pools are seasonal wetlands that support annual plant communities tolerant of ephemeral inundation. Most of the ∼8000 ha of undeveloped land at Beale Air Force Base is vernal pool grassland habitat (Platenkamp 1998) and is regularly grazed by cattle. Riverbank and Modesto geological formation underlie these vernal pools. Both formations originate in the Pleistocene and are composed of a relatively small amount of granitic sand stratified on top of a dark red clay and silt. Deep, well-drained soils form an alluvium derived from largely granitic rock sources characterize the San Joaquin Series soil of this vernal pool landscape (O'Green 2015).

### Host identity and frequency of infection

In late May and June of 2012, we investigated Dodder infection of host plants within the three zones of a vernal pool. Vernal pool zones (centre, transition and edge) represent varying lengths of inundation time throughout the season. The centre is the deepest part of the pool where inundation is longest, the edge is the outer limits of the pool and least inundated, and the transition zone occurs between the centre and edge (Emery 2009). We randomly chose 15 vernal pools within an ∼20-ha section of Beale Air Force Base designated by Air Force staff as safe for research. Collection of percent cover and host species data determined whether Dodder was associated with a vernal pool zone. During the peak of the Dodder growing season, random 0.25 m^2^ samples in the centre, transition, and edge zones quantified each plant species percent cover in each of the 15 vernal pools. We recorded the number of plants from each species that were parasitized. For the two primary host species, *E. castrense* (Apiaceae) and *N. leucocephala* (Polemoniaceae), we assessed whether *C. howelliana* cover increased with host species dominance in the community (frequency dependent).

### Effects on plant community

A Dodder removal experiment tested the hypothesis that Dodder will affect plant species diversity in vernal pools. When Dodder began to germinate on 24 March 2013, paired 0.25 m^2^ plots were placed in the centre and at the transition zones in each of the 15 vernal pools. The pools were chosen at random among pools known to support Dodder within the 20-ha section of Beale Air Force Base. In one of the each paired plots we manually removed germinating Dodder. We removed seedlings once a week until there were no emerging seedlings. Lack of cotyledons and orange colour easily distinguishes Dodder seedlings from other plants. If seedlings reached a host plant and develop any haustoria connections, we severed the connections to allow the host plant to develop normally (Pennings and Callaway 2002). In the other of the paired plots, germinating Dodder individuals grew and acquired a host without interference.

*Cuscuta howelliana* did not inhabit the edges of vernal pools, so we did not incorporate edge zone in the manipulative portion of this study. We placed each plot pair 0.5 m apart at the same elevation within a pool to obtain the best approximation of equal abundance and richness of plant species initially. Plant species richness (number of species; using Baldwin *et al*. 2012) and percent cover were measured in each 0.25 m^2^ plot every 1–2 weeks. By placing a 10 × 10 grid over each 0.25 m^2^ paired plot, percent cover is the number of grid squares covered by any one species (Meese and Tomich 1992). Sampling ceased once *C. howelliana* completed flowering at the end of May.

### Statistical analysis

The zone association study used analysis of variance (ANOVA) with *C. howelliana* cover as a dependent variable and zones (centre, transition and edge) as the independent variable. To test for difference among the zones, we used a post hoc test with a Bonferroni correction. We investigated whether the use of those host plant species was frequency dependent, percent cover of attacked hosts relative to their percentage of total plant cover using linear regression. A repeated-measures ANOVA analysed the Dodder removal experiment data. The removal and location (centre and transition zones) were fixed independent variables, and the dependent variables included total plant species richness, native plant richness, exotic plant richness, total percent cover of all non-*Cuscuta* plants and each plant species percent cover within all plots sampled. Even after log transformation, all data violated the sphericity assumption (Mauchley's test, *P* < 0.05), and therefore a Huynh–Feldt correction was used on all analyses.

To assess species composition in the removal study, analysis of similarity (ANOSIM) determined whether species composition was significantly different among treatments at each of the sampling periods. When appropriate (statistically significant ANOSIM), we conducted a similarity of percentages (SIMPER) to determine which species contributed to composition differences. We developed a non-metric multidimensional scaling (NMDS) plot that graphically presents differences in community composition based on Bray–Curtis differences. ANOSIM, SIMPER and NMDS were conducted using PAST version 1.94b (Hammer *et al*. 2001). All other analyses were conducted with SPSS version 21 (IBM Corp. 2012).

## Results

### Host identity and frequency of infection (Year 1)

Within the 15 vernal pools sampled, *C. howelliana* had a mean percent cover of 5.07 % (±standard deviation = 3.19 % and range = 0.4–11 %). *Cuscuta howelliana* had high percent cover in the centre zone (mean percent cover = 4.93, SD = 1.49) and transition zone (mean percent cover = 8.10, SD = 1.79) compared with the edge zone (mean percent cover = 0.47, SD = 0.47) (*F*_2,44_ = 7.791, *P* = 0.001). Of the 1365 parasitized plants, *E. castrense* and *N. leucocephala* together made up 84.3 %, of all the individual plants parasitized by *C. howelliana. Eryngium castrense* comprised 61.35 % of the percent cover of *C. howelliana*, and *N. leucocephala*, as a host, comprised 28.6 % of the percent cover of *C. howelliana*. Of the 32 plant species observed (Table 1), *C. howelliana* parasitized 16 of them. We used the Calflora database (www.calflora.org) to determine native, wetland generalist or non-native species status of each species and found that Dodder parasitism was associated with vernal pool endemics (versus wetland generalists or non-native species; *χ*^2^= 4.8, df = 2, *P* = 0.028).

**Table 1.** Catalogue of all plant species observed in 2012 and associated data on parasitism by *C. howelliana*, native status in California, and growth habit. A, annual, P, perennial, B, biennial.

Species	Family	Parasitized	Native	Habit
*Alopecurus saccastus*	Poaceae	No	Yes	A
*Agrostis avenacea*	Poaceae	No	No	P
*Aira caryophyllea*	Poaceae	Yes	No	A
*Briza minor*	Poaceae	No	No	A
*Lolium multiflorum*	Poaceae	No	No	A
*Polypogon monspeliensis*	Poaceae	Yes	No	A
*Taeniatherum caputmedusae*	Poaceae	No	No	A
*Fescuta bromoides*	Poaceae	No	No	P
*Eleocharis macrostachya*	Cyperaceae	Yes	Yes	P
*Juncus bufonius*	Juncaceae	No	Yes	P
*Brodiaea minor*	Asparagaceae	No	Yes	P
*Isoetes orcuttii*	Isoeteaceae	Yes	Yes	P
*Eryngium castrense*	Apiaceae	Yes	Yes	B
*Centromadia fitchii*	Asteraceae	Yes	Yes	A
*Lasthenia glaberrima*	Asteraceae	Yes	Yes	A
*Lasthenia fremontii*	Asteraceae	Yes	Yes	A/P
*Layia fremontii*	Asteraceae	No	Yes	A
*Leontodon saxatilis*	Asteraceae	Yes	No	A
*Psilocarphus brevissimus*	Asteraceae	Yes	Yes	A
*Plagiobothrys stipitatus*	Boraginaceae	Yes	Yes	A
*Plagiobothrys leptocladus*	Boraginaceae	No	Yes	A
*Downingia bicornuta*	Campanulaceae	Yes	Yes	A
*Cuscuta howelliana*	Convolvulaceae	No	Yes	A
*Elatine rubella*	Elatinaceae	No	Yes	A
*Croton setigerus*	Euphorbiaceae	No	Yes	A
*Trifolium dubium*	Fabaceae	No	No	A
*Erodium botrys*	Geraniaceae	No	No	A
*Lythrum hyssopifolia*	Lythraceae	Yes	No	A/P
*Navarretia leucocephala*	Polemoniaceae	Yes	Yes	A
*Ranunculus aquatilis*	Ranunculaceae	Yes	Yes	P
*Castilleja campestris*	Orobanchaceae	Yes	Yes	A
*Mimulus tricolor*	Phrymaceae	No	Yes	A

**Figure 1.**
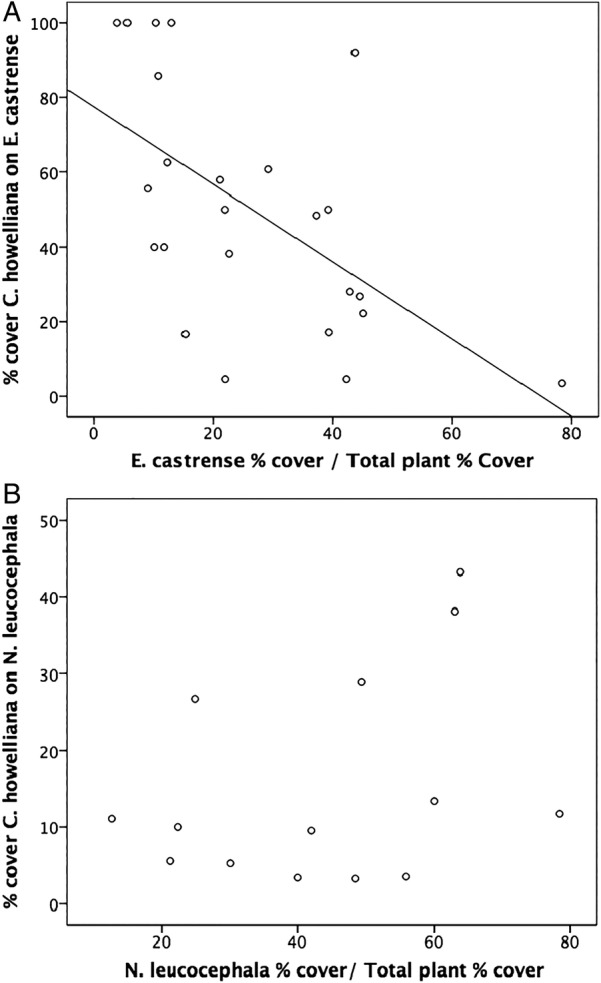
Scatterplot of (A) *E. castrense* covered by *C. howelliana* when *E. castrense* cover is standardized by total plant cover, and (B) *N. leucocephala* covered by *C. howelliana* when *N. leucocephala* cover is standardized by total plant cover.

Percent cover of Dodder on *E. castrense* declined linearly as the percent cover of *E. castrense* increased (*R*^2^ = 0.33, *F*_1,23_ = 10.95, *P* = 0.003; Fig. 1A). When *E. castrense* was rare in the community, it was parasitized to a greater degree. However, there was no relationship found with *N. leucocephala* (*R*^2^ = 0.12, *F*_1,23_ = 1.6, *P* = 0.230; Fig. 1B).

### Effects on plant community (Year 2)

**Figure 2.**
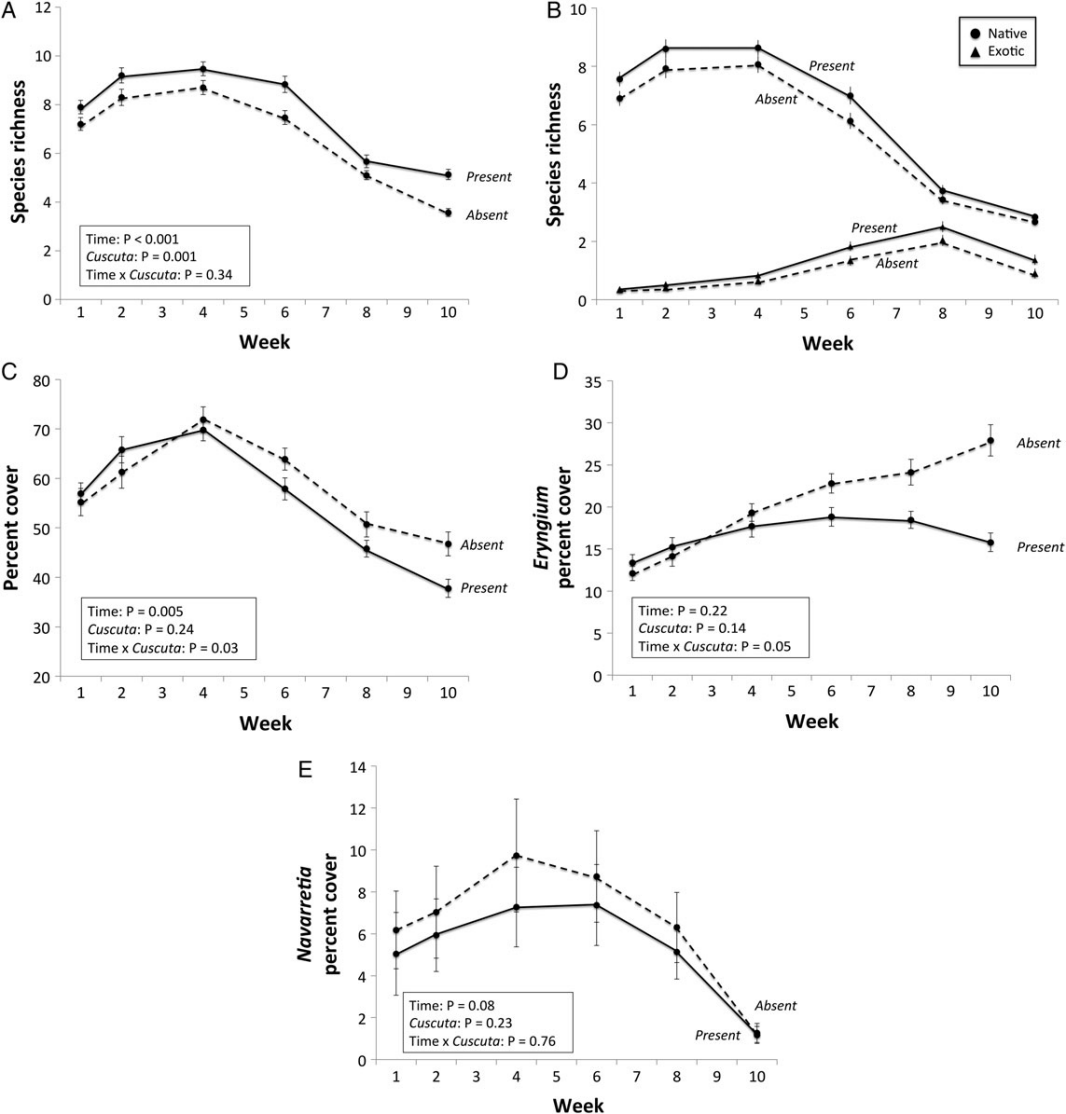
Mean (±SE) of (A) species richness, (B) native and exotic species richness, (C) total plant percent cover, (D) *E. castrense* cover and (E) *N. leucocephala* cover in *Cuscuta* present and removed treatments over time. Note that (C–E) present percent cover as the dependent variable and are all on different scales.

There were 29 total plant species found within all plots of the 15 vernal pools. However, average species richness per vernal pool ranged from 4 to 10 species over the course of the study (Fig. 2A). In plots with *C. howelliana*, total plant species richness was significantly greater (time × treatment: *F*_4.61, 258.39_ = 2.83, *P* < 0.001), ranging from 10 % higher in late March to 30 % higher in late May, compared with plots with removal plots (Fig. 2A, Appendix). Similarly, native and exotic plant species richness was greater with Dodder (Fig. 2B, Appendix). The timing in response to removal treatments differed between native and exotic species: native species richness increased during the first half of the experiment, and exotic species increased during the latter half of the experiment (Fig. 2B). Total plant cover increased in response to Dodder removal over time (time × treatment: *F*_3.66, 190.38_ = 2.83, *P* = 0.03; Fig. 2C, Appendix). However, there were no effects of zone (centre and transition) on plant species richness and cover.

Dodder removal increased *E. castrense* percent cover, a frequently parasitized host (time × treatment: *F*_3.36, 174.79_ = 16.61, *P* < 0.001; Fig. 2D, Appendix)*.* From early March to the end of April, the *E. castrense* percent cover was not different between the treatments. Beginning in late April, the *E. castrense* percent cover began to decrease in the Dodder plots and increase in the Dodder removal plots. At the end of May, *E. castrense* cover was 43 % higher in the Dodder removal plots compared with the Dodder plots (Fig. 2D). There was no effect of zone (centre and transition) on *E. castrense* percent cover (Appendix).

The removal treatment did not affect percent cover of *N. leucocephala*, another of the frequent hosts of *C. howelliana* (*F*_3.12, 162.32_ = 0.32, *P* = 0.816; Appendix), but *N. leucocephala* cover did differ among zones (Appendix). In both experimental plots, percent cover *N. leucocephala* ranged from 0 to 50 % during the first five sampling periods. During the last sampling period, the majority of the *N. leucocephala* had finished its life cycle (Fig. 2E). All other plant species observed did not have significant cover differences between Dodder removal and control plots.

**Figure 3.**
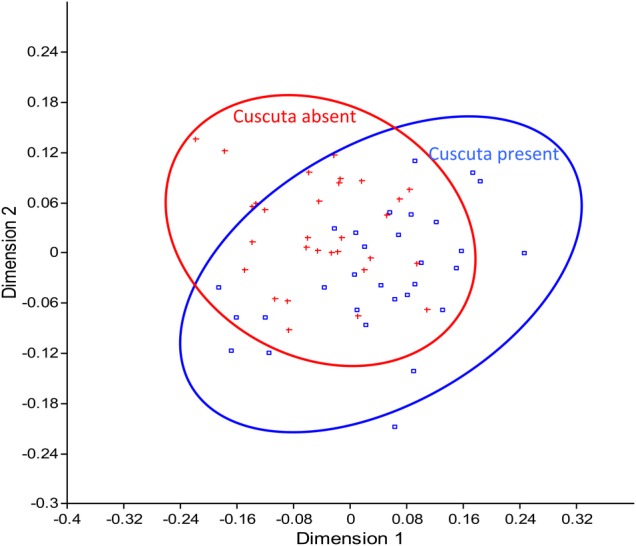
Non-metric multidimensional scaling (NMDS) plot of communities in *Cuscuta* present and removed plots with 95 % concentration ellipses. Scaling was based on Bray–Curtis similarity distances.

Plant species composition between treatments differed only during the last sampling period according to the ANOSIM results (*R* = 0.3448, *P* < 0.01). During this sampling period, SIMPER indicated that *E. castrense* (38 %) contributed most to differences between treatments, which increased with removal (Table 2). *Hemizonia fitchii* (26 %), *Mentha pulegium*, (10 %) and *Leontodon taraxicoides* (8 %) also contributed to the differences between treatments, but their cover all decreased with Dodder removal. The remaining nine plant species also decreased with removal, but altogether contributed <6 % (Table 2). The NMDS plot reflected these compositional differences between treatments (Fig. 3).

**Table 2.** SIMPER results from final sampling period comparing *C. howelliana* presence versus removal. Species are listed in descending order according to their contribution towards the difference in species composition between treatments. ^N^A California native and ^E^an exotic species.

Taxon	Contribution	Cumulative %	Mean abundance *C. howelliana* present	Mean abundance *C. howelliana* removal
*Eryngium castrense*^N^	15.62	38.15	15.8	27.9
*Hemizonia fitchii*^N^	10.69	64.26	13.1	11.7
*Mentha pulegium*^E^	3.946	73.9	2.9	2.03
*Leontodon taraxicoides*^E^	3.319	82.01	1.93	1.77
*Navarretia leucocephala*^N^	2.401	87.87	1.27	1.17
*Eleocharis macrostachya*^N^	2.387	93.7	1.1	1.5
*Eremocarpus setigerus*^N^	1.203	96.64	0.767	0.367
*Polypogon monspliensis*^E^	0.5808	98.06	0.3	0.233
*Lythrum hyssopifolium*^E^	0.3113	98.82	0.233	0.0333
*Lolium multiflorum*^E^	0.2621	99.46	0.133	0.0667
*Plantago lanceolata*^E^	0.1346	99.79	0.133	0
*Broidea elegans*^N^	0.04536	99.9	0.0333	0
*Linum bienne*^E^	0.04079	100	0.0333	0

## Discussion

The goal of this study was to evaluate the role of a parasitic plant *C. howelliana* on the plant species composition of California vernal pools. *Cuscuta howelliana* occurred in the centre and transition zones of vernal pools, and vernal pool endemics tended to be targeted: *E. castrense* and *N. leucocephala* were found to be the most frequent hosts. However, only *E. castrense* percent cover was negatively affected by Dodder. The shift in species composition suggested that *C. howelliana* reduced the dominance of *E. castrense*, which resulted in increased native and exotic species richness.

The two preferred host plant species only rarely occurred on the edge zones of vernal pools. *Eryngium castrense* and *Navarretia leucocephela* have previously been found in the centre, deeper areas of vernal pools (Bauder 2000; Emery *et al*. 2009). Host distribution is a possible explanation of why Dodder was only rarely observed in the edge zone. However, the restriction of Dodder to the deeper portions of a vernal pool could be due to other factors such as hosts' access to limiting nutrients (Pate *et al*. 1990) or preference for less-stressed hosts (Miller *et al*. 2003). *Navarretia leucocephela* have been found to increase reproduction in the absence of competitors, especially in the centre parts of vernal pools (Emery *et al*. 2009); this could also influence the nutrient availability for Dodder. This is consistent with past research on a congeneric, *Cuscuta europaea*, which parasitized hosts with higher nutrient availability (Kelly 1992).

Although there were 16 total plant species with haustoria connections, *E. castrense* and *N. leucocephala* were found to be the most frequent hosts of *C. howelliana*. Congenerics (*C. costaricensis*, *C. salina*) have also shown host preferences in their respective communities (Kelly *et al*. 1988; Pennings and Callaway 1996). The higher cover of Dodder when *E. castrense* was not a dominant in the community (Fig. 1A) suggested that even when there are many alternative hosts, that *E. castrense* is still parasitized to a greater degree. In contrast, Dodder cover did not increase with increased *N. leucocephala* cover, nor did it increase with Dodder removal. The differential effects on the two common host species may result from differences in susceptibility or life histories: *E. castrense* is a larger (20–60 cm; Preston *et al*. 2013) biennial plant, while *N. leucocephala* is a smaller (3–15 cm; Johnson 2013) annual plant. *Eryngium castrense*, as a biennial, also has a longer period of growth during the season than the annual *N. leucocephala*. The use of a biennial host may increase Dodder's growth by ensuring a stable resource over the growing season and not expend energy continuously foraging for other suitable hosts.

Plant species composition was significantly different with Dodder removal during only the last sampling period. *Eryngium castrense* contributed the greatest to differences in plant species composition. This is also reflected in *E. castrense* percent cover increasing over time with Dodder removal. Despite a long list of known factors and relationships at work in the complex ecological network in California vernal pools, there is less understanding of species interactions in maintaining vernal pool richness. The keystone predator concept posits that consumption of a competitively dominant prey can increase species richness or change the species composition of an ecosystem (Paine 1966; Mills *et al*. 1993). *Eryngium castrense* is a common vernal pool plant species (Barbour *et al*. 2005), but there are no studies quantifying its competitive ability. Therefore, we cannot say for certain that the mechanisms for keystone predation are at play here. However, few field studies have conducted removal experiments to test for keystone species effects (Valls *et al*. 2015). This study provides evidence that Dodder plays this role with vernal pool plant species richness and composition. *Cuscuta howelliana* increased plant species richness, consistent with what is expected from the effects of a keystone predator. By this mechanism, more habitat and resources was likely made available for other plant species to become established.

*Cuscuta howelliana* effects were similar to *C. salina*, which occurs in coastal salt marshes and parasitizes the competitively dominant *Salicornia virginica* (Pennings and Callaway 1996). Due to lower plant species diversity and higher competitive dominance of *S. virginica*, the effect of *C. salina* plays a larger role in providing habitat for competitively inferior plant species (Pennings and Callaway 1996). Specifically, at certain elevations within the salt marsh, *S. virginica* will achieve a near monoculture in the absence of *C. salina. Cuscuta salina* resets succession as *S. virginica* patches die following infection (Pennings and Callaway 1996). Our results suggest a similar mechanism in vernal pools, and it would be interesting to examine the consequences of these interactions on species composition patterns at different spatial and temporal scales. The annual inundation–desiccation cycle and dominance of annual species make vernal pools very different from coastal marshes and may result in different spatiotemporal dynamics.

The composition of the vernal pool communities also included seven exotic species: *Polypogon monspeliensis*, *Lythrum hyssopifolia*, *Lolium multiflorum*, *M. pulegium*, *L. taraxicoides*, *Plantago lanceolata* and *Linum bienne*. The percent cover of these species increased, on average, with Dodder (Table 2). While increases in species cover were not significant, the observed trends do suggest the potential for facilitation of exotic species into vernal pools by Dodder. Further, exotic species richness did significantly increase with *C. howelliana* during the latter half of the experiment. The ‘enemy release hypothesis’ has been identified in various ecosystems with a diversity of pathogens, parasites, and predators (Colautti *et al*. 2004; Liu and Stiling 2006; Heard and Sax 2013). This mechanism for species invasions is the result of the absence of herbivores regulating exotic species. We know of no situation where parasitic plants have acted as the agent for exotic plant facilitation and, therefore, the present study identifies a potential novel mechanism for exotic species invasion.

The climate in California varies tremendously across years, which strongly contributes to plant community structure (Pitt and Heady 1978; Hobbs and Mooney 1995). These studies occurred during below-normal rainfall years, which could have influenced our results in at least two ways. First, the amount and timing of rainfall can affect species composition of California vernal pools (Bliss and Zedler 1997; Collinge *et al*. 2011). Drought can increase invasion of exotic species into vernal pools (Collinge *et al*. 2011), which may dilute the species pool of potential hosts. Therefore, changes to species richness and relative abundance could influence the effects of Dodder. Second, different environmental conditions can affect host–parasite interactions and co-evolution (Vale *et al*. 2008; Wolinska and King 2009). Rainfall specifically alters host–parasite interactions. For example, Blue Palo Verde (*Cercidium floridum*) mortality correlates with Desert Mistletoe (*Phoradendron californicum*) infestation during severe drought in the Mojave Desert (Spurrier and Smith 2007). Therefore, Dodder could interact with hosts and the vernal pool plant community differently depending on precipitation.

*Cuscuta howelliana* is another example of a parasitic plant that has a positive effect on plant species richness by parasitizing an abundant host. However, this also led to an increase in exotic species richness and cover. In other *Cuscuta* species (e.g. *C. salina*), individuals can parasitize multiple individuals simultaneously and affect species richness (e.g. Grewell 2008). Our study design did not attempt to identify this mechanism, but this could potentially be important for diversity maintenance. This research contributes to the development of a more complete understanding of how California vernal pools maintain biodiversity, and how parasitic plants interact with the communities around them.

Considering the rampant loss of vernal pool habitat in recent years (Holland and Jain 1988; Holland 1998), the effect of Dodder is another important consideration when trying to maintain or increase species richness in a managed vernal pool habitat. Inclusion of Dodder in created or mitigated pools could function to maintain a higher level of plant species richness over time. However, this increase in species richness also includes an increase in exotic species cover. Nonetheless, the interactions among species, including parasitic plants, needs to be considered in restoration and management of California vernal pools.

## Conclusions

This research contributes to the body of knowledge that parasitic plants are important to the maintenance of species diversity in communities (Pennings and Callaway 2002; Hatcher *et al*. 2012) and can act as a keystone predator. Long-term research that incorporates the climatic variation with the spatiotemporal effects of *C. howelliana*, Boggs Lake dodder, on its associated vernal pool plant species would be important to predict their long-term effects. A broader perspective that includes the effects of multiple trophic levels (e.g. other parasites, herbivores, pollinators) will further our understanding of these imperiled communities. Furthermore, a holistic approach that includes species interactions with climatic variation will strengthen the management, conservation and restoration of California vernal pools.

## Sources of Funding

The study was supported by a California Native Plant Society grant.

## Contributions by the Authors

Submitting authors conceived, designed and executed this study and wrote the manuscript.

## Conflict of Interest Statement

None declared.

## Appendix

Within-subject effects of repeated-measures general linear model (Huynh–Feldt correction) for a dependent variable of (1) plant species richness, (2) native species richness, (3) exotic species richness, (4) total plant percent cover, (5) *Eryngium castrense* percent cover and (6) *Navarretia leucocephala* percent cover. Asterisks indicate: **P* < 0.05, ***P* < 0.01, ****P* < 0.001.

Plant species richness

**Table:**

Source	df	*F*	*P*
Time	4.61, 258.39	180.86	**<0**.**001**
Time × zone	4.61, 258.39	2.39	0.043
Time × treatment	4.61, 258.39	5.72	**<0**.**001**
Time × zone × treatment	4.61, 258.39	0.93	0.457

Native richness

**Table:**

Source	df	*F*	*P*
Time	4.28, 235.62	229.73	**<0**.**001**
Time × zone	4.28, 235.62	0.62	0.663
Time × treatment	4.28, 235.62	2.89	**0**.**020**
Time × zone × treatment	4.28, 235.62	1.95	0.098

Exotic richness

**Table:**

Source	df	*F*	*P*
Time	3.82, 209.81	76.61	**<0**.**001**
Time × zone	3.82, 209.81	3.77	**0**.**006**
Time × treatment	3.82, 209.81	2.86	**0**.**027**
Time × zone × treatment	3.82, 209.81	0.47	0.750

Total plant percent cover

**Table:**

Source	df	*F*	*P*
Time	3.66, 190.38	49.27	**<0**.**001**
Time × zone	3.66, 190.38	2.95	**0**.**025**
Time × treatment	3.66, 190.38	2.83	**0**.**030**
Time × zone × treatment	3.66, 190.38	0.33	0.842

*E. castrense* percent cover

**Table:**

Source	df	*F*	*P*
Time	3.36, 174.79	35.09	**<0**.**001**
Time × zone	3.36, 174.79	1.06	0.374
Time × treatment	3.36, 174.79	16.64	**<0**.**001**
Time × zone × treatment	3.36, 174.79	0.43	0.753

*N. leucocephala* percent cover

**Table:**

Source	df	*F*	*P*
Time	3.12, 162.32	13.55	**<0.001**
Time × zone	3.12, 162.32	2.82	**0.039**
Time × treatment	3.12, 162.32	0.32	0.816
Time × zone × treatment	3.12, 162.32	0.45	0.723
